# Chemical Composition of Fat Bloom on Chocolate Products Determined by Combining NMR and HPLC–MS

**DOI:** 10.3390/molecules29133024

**Published:** 2024-06-26

**Authors:** Lena Trapp, Niels Karschin, Markus Godejohann, Hilke Schacht, Hermann Nirschl, Gisela Guthausen

**Affiliations:** 1 Institute of Mechanical Process Engineering and Mechanics, Karlsruhe Institute of Technology, 76131 Karlsruhe, Germany; 2 Bruker BioSpin GmbH & Co. KG, 76275 Ettlingen, Germany; 3 Fraunhofer Institute for Process Engineering and Packaging IVV, 85354 Freising, Germany; 4 Engler-Bunte-Institut, Water Chemistry and Technology, Karlsruhe Institute of Technology, 76131 Karlsruhe, Germany

**Keywords:** fat bloom on chocolate, NMR spectroscopy, HPLC–MS, MRI, process control, quality assurance

## Abstract

To reduce unwanted fat bloom in the manufacturing and storage of chocolates, detailed knowledge of the chemical composition and molecular mobility of the oils and fats contained is required. Although the formation of fat bloom on chocolate products has been studied for many decades with regard to its prevention and reduction, questions on the molecular level still remain to be answered. Chocolate products with nut-based fillings are especially prone to undesirable fat bloom. The chemical composition of fat bloom is thought to be dominated by the triacylglycerides of the chocolate matrix, which migrate to the chocolate’s surface and recrystallize there. Migration of oils from the fillings into the chocolate as driving force for fat bloom formation is an additional factor in the discussion. In this work, the migration was studied and confirmed by MRI, while the chemical composition of the fat bloom was measured by NMR spectroscopy and HPLC–MS, revealing the most important triacylglycerides in the fat bloom. The combination of HPLC–MS with NMR spectroscopy at 800 MHz allows for detailed chemical structure determination. A rapid routine was developed combining the two modalities, which was then applied to investigate the aging, the impact of chocolate composition, and the influence of hazelnut fillings processing parameters, such as the degree of roasting and grinding of the nuts or the mixing time, on fat bloom formation.

## 1. Introduction

The chemical composition of fat bloom has been studied so far by HPLC (high pressure liquid chromatography) [1,2], which allows the separation of the molecules contained in the sample, but not a direct and biunique identification of the chemical substances and determination of the molecular composition. Discussions about the mechanisms causing fat bloom converge to the conception that migration of liquid fats needs to be taken into account [3]. As fat crystallization in and on chocolate is strongly influenced by the fat composition [4], fillings and their fat composition need to be considered.

Usually, fats in chocolate contain triacylglycerides (TAGs), which are composed of fatty acids (FAs) that are covalently bound to glycerol. The TAGs that make up the fat differ according to the type and position of the three FAs esterified with glycerol. In the following, the abbreviation of the TAG is derived from the indicated letters of the three FAs they contain. Major constituents are POS (1-Palmitoyl-2-oleoyl-3-stearoyl-glycerol), SOS (1,3-Distearoyl-2-oleoyl-glycerol), and POP (1,3-Dipalmitoyl-2-oleoyl-glycerol) [1]. Examples of minor constituents are OOO (1,2,3-Trioleoyl-glycerol), PLP (1,3-Dipalmitoyl-2-linaloyl-glycerol) and POL (1-Palmitoyl-2-oleoyl-3-linaloyl-glycerol) [1]. In contrast, hazelnut oil mainly consists of OOO and LOO (1-Linaloyl-2,3-dioleoyl-glycerol) [2], leading to the liquid state of the oil at room temperature. Further constituents of hazelnut oil are, for example, POO (1-Palmitoyl-2,3-dioleoyl-glycerol) and SOO (1-Stearoyl-2,3-dioleoyl-glycerol) [2]. Ranges of melting temperature and possible crystal forms in the solid state are given in the literature [5] and are summarized in Table 1 for some of the TAGs contained in chocolate or fillings, with the intention to give a first impression of the pure phases of the involved fats. We are aware of the fact that fat compositions need not behave like a pure fat but may deviate for that specific state significantly.

Little is known about the fat bloom’s chemical composition and its dependence on the overall chocolate composition, including the fillings, especially in pralines. Nut-based fillings are thought to cause a more pronounced fat bloom. Milk fats, sugars, emulsifiers and other fillings need also to be considered [15]. Fat blooming is thought to be due to crystallization of TAG at the surface of chocolate, while the question about its driving forces needs to be answered. The hypothesis is that the mobile fats in the fillings migrate into the chocolate shell and dissolve the fats contained therein. The fats together migrate to the surface and crystallize there in a mixed crystalline form of TAG. To clarify the details of the commonly occurring, but in detail rather complex, processes, an approach could be to study the chemical composition of the fat bloom and compare it with that of the mobile migrating oils, for example, in hazelnuts or hazelnut oil. Another approach is to study the composition of shells and fillings of differently produced pralines as a function of storage time and to quantify the changes in structure and fat bloom composition. A short overview of the investigated pralines and chocolates is given in Table 2, the details are described in Section 4.1. Samples.

NMR (nuclear magnetic resonance) spectroscopy, in the form of ^1^H NMR and ^13^C NMR, is a well-known analytical tool to investigate chemical structure and composition, and the relative concentrations can at least be estimated if not accurately quantified even in complex mixtures. In addition, 2D NMR has the capability to detect connections between different structural functional groups. Additionally, the identification of specific compounds can be facilitated by comparison with chemical standards or spiking. NMR and its imaging version MRI (magnetic resonance imaging) work non-destructively and therefore allow for longitudinal studies of one and the same object or sample. However, NMR-based quantification of lipids is typically limited by signal overlap, especially in ^1^H NMR spectra. This fact is evident for the CH_2_ groups in the FA, which are linked to the chemical structure but can hardly be distinguished in the ^1^H NMR spectra. In contrast, ^13^C NMR has a much lower sensitivity due to the low gyromagnetic ratio combined with the low natural abundance of ^13^C nuclei, but it offers much better signal dispersion thanks to the larger chemical shift dispersion and the absence of spin–spin couplings.

In mass spectrometry (MS), where the sensitivity strongly depends on the ionization efficiency of analytes in the ion source, detection limits are typically much lower when compared to NMR. However, quantification relies on external or internal calibration with pure reference compounds and therefore the availability of standards. High resolution MS allows the determination of the molecular formula of organic compounds. Furthermore, isolation of precursor ions followed by collision induced dissociation (CID) combined with high resolution mass detection helps to determine the molecular formula of fragments, which is especially helpful in the case of TAG analysis. Special chromatography techniques are necessary, however, to separate regio- or enantiomeric isomers of isobaric TAG [16,17]. The combination of MS, ^13^C and ^1^H NMR, however, allows for a comprehensive description of the chemical composition of the TAG involved in fat bloom.

## 2. Results

Measurements have been performed on pralines and commercially available chocolate. Firstly, a longitudinal study on self-made pralines revealed the effect of aging and the influence of chocolate processing parameters, and secondly, commercially available chocolates with yoghurt and nougat fillings were studied. The results are structured starting with exemplary MR images of pralines, followed by NMR spectroscopy and HPLC–MS.

### 2.1. MRI on Pralines

**Figure 1:**
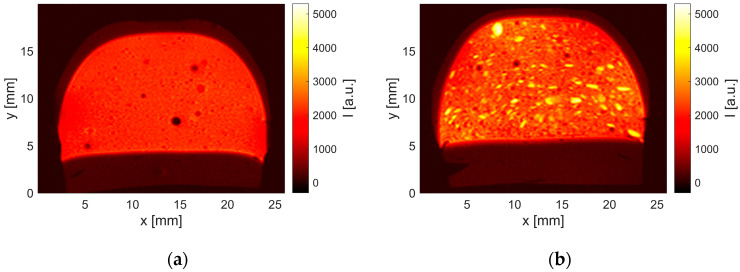
MR image of a fresh praline with a (**a**) fine ground (praline 1) and (**b**) coarse ground filling (praline 12), revealing typical transverse relaxation properties of the constituents. Nut pieces appear with signal intensities of 4000–5000 a.u., the filling is in the range of 2500 a.u., while the shell appears with signal intensities of 500–1000. The surrounding of the praline and small air bubbles in the filling have signal intensities on the noise level well below 120 a.u., when using the MRI parameters in Table 9.

MRI was used as an imaging technique to investigate the aging processes inside the praline without destroying it. Compared to other imaging methods, MRI offers the possibility of imaging the chemical compositions within the sample in a spatially resolved manner. Opaque media, such as a praline, are no problem. MR images of the self-made pralines show their structure (Figure 1). Similar results were measured for the pralines with regard to aging. Examples of this are shown for praline 1 (P1 in Table 2) and praline 12 (P12 in Table 2). The nut-based filling exhibits comparatively long transverse relaxation, which is typical for liquid oils and appears in larger signal intensities in the images. In contrast, the chocolate shell shows shorter transverse relaxation times leading to smaller signal intensities. The shell thereby is hardly visible. Air bubbles in the filling appear as black spots. The difference in grind size of the nuts contained in the filling is evident when comparing the images in Figure 1a,b. Larger nut pieces in praline 12 (Figure 1b) can be distinguished from the rest of the filling by higher signal intensities (4000–5000 a.u.).

**Figure 2:**
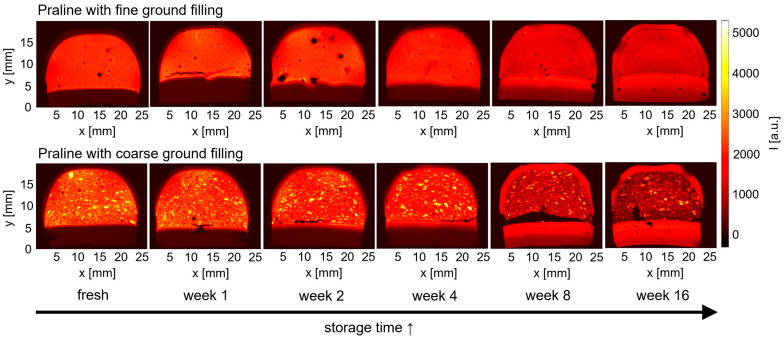
MR images of pralines with a fine ground (praline 1, top) and coarse ground (praline 12, bottom) filling in the longitudinal study (storage at 20 °C). The pralines were removed from storage and measured after storage times of 1, 2, 4, 8 and 16 weeks. Between the fourth and eighth week in particular, the signal intensity of the chocolate shell increases, while it decreases for the filling.

The appearance of the pralines in the MR images changes with aging (Figure 2): the shell shows increasing signal intensity indicating an increasing concentration of liquid components, while the filling loses signal intensity with time. A spatial separation of the filling and chocolate layer was observed on the bottom during storage, particularly in pralines with a coarse ground filling (praline 12). In all images, small air bubbles are visible with a distribution of volumes and positions in the fillings as well as in the shells, similar to the findings in Figure 1.

Taking a closer look at the shells as a function of storage time, the increase of signal intensity is evident for both praline types. In addition, the praline’s bottom shell shows an internal structure, especially in the images after 8 and 16 weeks of storage. It is composed of two layers and shows up with two distinct intensities. Rather than the expected continuous gradient, two regions were observed, which leads to the conclusion that a migration barrier between the two parts of the shell is present. It appears more like a migration front rather than a diffusive, spread out transition of the fat component, delivering a bright MR signal.

### 2.2. NMR Spectroscopy for Identification of TAG

**Figure 3:**
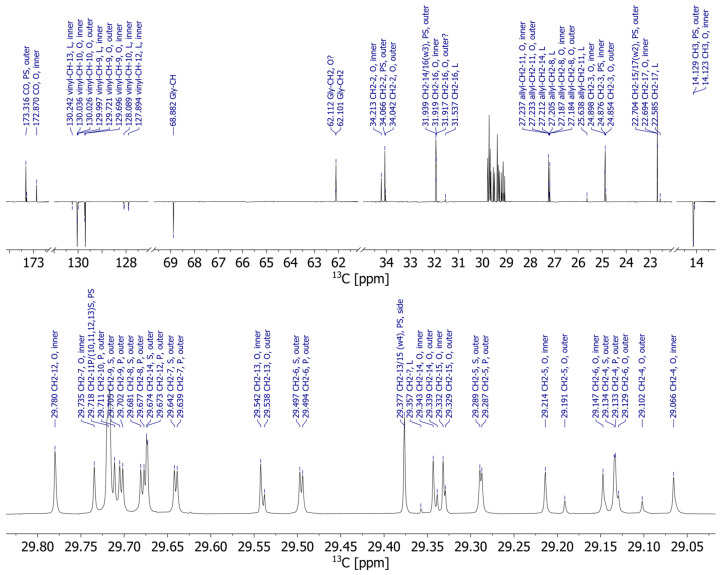
^13^C-APT spectrum (APT: Attached Proton Test) of fat bloom of praline 5 (fine ground, intermediate mixing time of 30 min at 40 °C) measured at a ^13^C Larmor frequency of 201 MHz (**top**) and a zoom-in of the spectral region 29.83 ppm–29.02 ppm (**bottom**). At this field of 18.8 T, nearly all resonances are resolved, and via 2D NMR almost all peaks could be assigned (large letters P, O, L, S; position of the FA in the TAG: inner, outer together with the resonances’ chemical shift; tentative assignments marked with?). Notice the distinct resonances for a number of lines of inner and outer oleic acid (O). In particular, 4-CH_2_, 5-CH_2_ and 6-CH_2_ can be resolved to the baseline (zoom-in spectrum, bottom).

**Figure 4:**
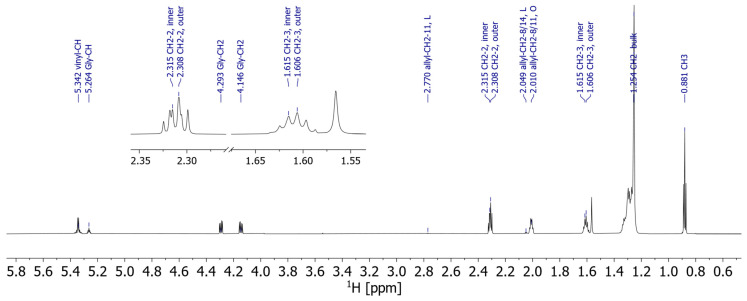
800 MHz ^1^H spectrum of the fat bloom of praline 5. The glycerol ^1^H, the allylic and vinylic ^1^H, the methyl group, and the 2-CH_2_ and 3-CH_2_ groups are resolved and assigned, and everything else is found in a bulk peak around 1.3 ppm. However, the inner and outer 2-CH_2_ and 3-CH_2_ can be distinguished (insert).

The MR images indicate selective fat migration, and the question needs to be addressed as to whether the fat composition of the fat bloom at the outer surface of the chocolate shell shows hints of these migrating components from the filling. Therefore, we per-formed NMR spectroscopy on the shell and the fillings, on raw materials such as cocoa butter and hazelnut oil, and on the fat bloom that had formed on the surface. The fat bloom of the pralines appearing in the NMR spectra (exemplarily for the fat bloom of praline 5 ^13^C NMR spectra in Figure 3 and ^1^H NMR spectra in Figure 4) is composed of approximately equal parts of stearic acid (S), palmitic acid (P), and oleic acid (O), with small amounts of linoleic acid (L). More importantly, the same FA can be substituted either at one of the outer 1/3-OH groups or at the inner 2-OH group of the glycerol core as measured in the ^13^C NMR spectrum (Figure 3). The FA’s 2-CH_2_ and 3-CH_2_ are close enough to the glycerol, so that the inner and outer acids are separated in ^1^H NMR (Figure 4), and the assignment has been performed by HMBC.

**Figure 5:**
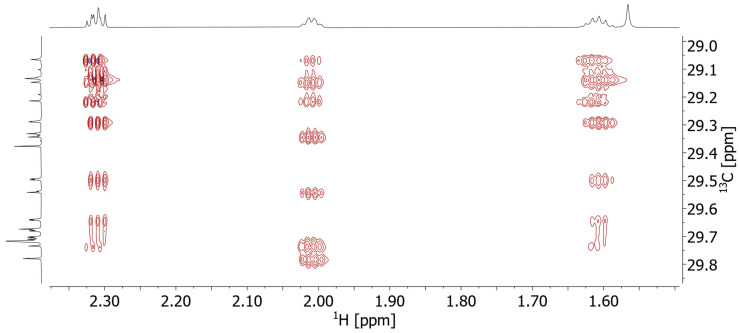
Zoom of the 800 MHz HSQC-TOCSY spectrum (100 ms mixing time) of the fat bloom of praline 5. The cross peaks of 2-CH_2_ and 3-CH_2_ (2.3 ppm and 1.6 ppm, respectively) vary slightly in ^1^H shift depending on the substitution position on the glycerol center (inner/outer, see also Figure 3 and Figure 4). Careful analysis leads to the conclusion that O (oleic acid) is predominantly substituted at the inner position, while P and S (palmitic and stearic acids) are mostly substituted at the outer positions.

HSQC–TOCSY (Heteronuclear Single Quantum Coherence–TOtal Correlation SpectroscopY) reveals correlations between various ^13^C resonances with these 2-CH_2_ and 3-CH_2,_ and in that way the corresponding FAs have been assigned to their substitution position on glycerol (Figure 5). The HSQC–TOCSY is uniquely useful for these samples: the TOCSY mixing allows transfer of ^1^H magnetization across long chains of unresolved CH_2_-^1^H to the well-dispersed carbon dimension and consequently allows us to distinguish these CH_2_ groups and assign them to a specific FA.

The substitution pattern in FA at glycerol in the fat bloom turned out to be rather well-defined: the saturated acids were exclusively substituted in the outer position, while the unsaturated O was primarily substituted at the inner position, although we could detect a minor set of resonances originating from O substituted at the outer glycerol–OH. We conclude that the praline fat bloom is mostly composed of POP, POS, and SOS, with a minor fraction potentially being OOO. The exact amount of OOO is the only measurable difference between the various praline samples (1–12), and the spectra are otherwise remarkably similar. The resonance assignment was corroborated by spectra of pure standards of the four aforementioned TAGs, which were commercially available.

These findings are in line with what is known about the composition of cocoa butter [18,19], and the acquired spectra of pure cocoa butter align very well with those of the fat bloom. The notable difference is that the praline fat bloom has an up to fivefold higher content of outer O, indicating OOO. A similar trend was observed for the resonances of L: L in cocoa butter is almost exclusively substituted at the inner position (XLX, X being an arbitrary FA), while in the fat bloom there is a measurable quantity of L substituted at the outer position (LXX, LXL). These TAG with unsaturated FA on the outer OH of glycerol are known to occur in hazelnut oil, which contains only around 10% saturated FA according to the label of the commercial oil. NMR of hazelnut oil confirms a high content of unsaturated FA (O/L) at the outer position. In contrast, the spectra of extracts of the raw chocolate shell do not show a significant amount of outer unsaturated FA, and are essentially indistinguishable from the spectra of cocoa butter.

**Figure 6:**
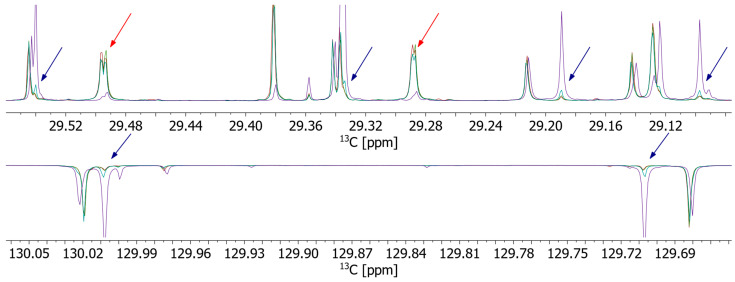
Overlay of ^13^C-APT spectra of cocoa butter (red), hazelnut oil (violet), and fat bloom of commercial yoghurt (green) and nougat (cyan) chocolates in the two spectral regions around 29.33 ppm (**top**) and 129.85 ppm (**bottom**). Overall, fat bloom spectra are very similar to cocoa butter spectra. However, the spectra of the nougat fat bloom show increased intensity for outer O, which is a main component of hazelnut oil. These peaks are marked by violet arrows. Two other groups, assigned to P and S in outer Figure 3, show up in fat bloom and cocoa butter (marked by red arrows), but not in hazelnut oil.

Additionally, we compared fat bloom very carefully when harvested from commercial chocolates with and without hazelnuts (nougat and yoghurt, Figure 6). While the fat bloom from the nut-free chocolate (yoghurt) is again remarkably similar to pure cocoa butter, the fat bloom from nougat chocolate shows an increased concentration of outer unsaturated FA, very similar to the fat bloom of the pralines. This finding indicates the migration of hazelnut oil components to the chocolates’ surface and the formation of mixed crystals in fat bloom.

### 2.3. Mass Spectrometry on FA and TAG in Fat Bloom and Starting Material

**Figure 7:**
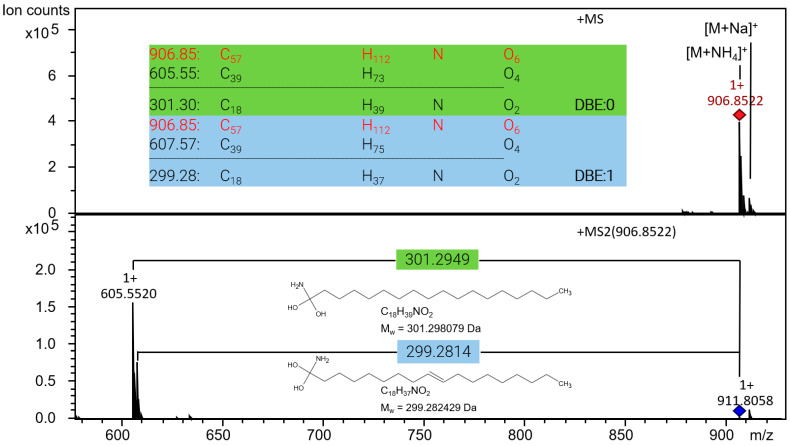
MS and MS/MS spectra exemplarily of SOS: After determination of the molecular formula of the ammonium adduct [M+NH_4_]^+^ of the TAG in the MS spectrum on top and the two resulting diacylglycerides [DAG]^+^ in the MS/MS spectrum below, the difference is calculated and corresponds to the neutral loss after CID, representing the FA as part of the TAG. The calculation of the double bond equivalent (DBE) of the loss finally reveals the number of double bonds present in the corresponding FA. Conventionally in MS spectra, *m*/*z* is specified as dimensionless.

MS is well known as a powerful and complementary analytical technique to NMR spectroscopy with a much larger sensitivity. It was applied to complete the study and to independently corroborate the chemical assignments made in NMR. Mass spectrometric investigations were divided in two parts:

1. Qualitative investigation of the type of TAG in the starting materials (hazelnut oil, nougat, cocoa butter and chocolate coating) and fat bloom of the pralines by HPLC–MS/MS. This also includes the calculation of relative ion count integrals for the TAG identified in the samples. Figure 7 exemplifies the interpretation of MS/MS spectra of the symmetrical TAG SOS.
2. Quantitative analysis of FA and TAG available as reference standards in the starting material and fat bloom samples using working standards for calibration as listed in Section 4.2.

The qualitative investigation resulted in the tentative identification and relative distribution of TAG in the samples. This tentative identification does not reflect the positional differentiation within the TAG, as this is a very time-consuming approach in LC–MS, and since this task can be accomplished by structure elucidation using NMR. In Table 3, the result of the tentative identification is compiled.

The relative distribution of the TAGs (Table 3) based on the ion count integral is shown in Table 4 and indicates that TAGs with a high degree of unsaturation are present in hazelnut oil and nougat, whereas TAGs with saturated FA form the fat bloom and are also present in the chocolate coating and cocoa butter of the pralines.

The quantitative analysis of FAs was performed in negative ionization mode, and the detection of TAGs was performed with positive ionization in full scan MS mode. Table 5 summarizes the results of the determination of FAs in the fat bloom obtained from the praline samples, which reveals only low fractions of FAs, mainly P and O. Table 6 lists the relative content of TAGs in the fat bloom of the praline samples based on the total weight of the fat bloom. The dominant TAG is POS, followed by POP and SOS.

## 3. Discussion

The results of MRI, NMR spectroscopy and MS provide comprehensive insights into fat blooming of filled chocolates. While MRI shows the migration of TAG from the filling to the chocolate shell and provides evidence for migration barriers, NMR spectroscopy and MS allow for the determination of chemical composition of the fat bloom and complement each other. When comparing the spectra of the different components (filling, initial and final chocolate shell, as well as fat bloom) with initial composition and reference standards, the migrating TAG can be identified, and as a result the migration process is described now in more detail.

In NMR spectroscopy, the unsaturated FAs from cocoa butter is distinguished from those from hazelnut oil due to the different substitution on the glycerol (inner and outer positions). Small, but reliably measurable and interpretable shift differences allow this very detailed assignments of TAG.

The composition of fat and oils from cocoa butter and hazelnut oil was thus found to be fundamentally different. Cocoa butter is mostly made of POP, SOS, and POS, with small amounts of PLP. The high fraction of saturated FA determines the appearance. Cocoa butter is solid, crumbly and almost brittle at room temperature. In contrast, hazelnut oil contains around 75% O, i.e., the TAG OOO is its main constituent. The second most common acid is L, found in TAGs such as OLO, OLL, and LLL. P, with a fraction below 10%, estimated at 6% in the NMR spectra and found mostly in PLO, is the most abundant saturated FA. As a consequence, hazelnut oil is liquid at room temperature.

The TAG with only unsaturated FAs are expected to be rather mobile, which causes increased MRI signal intensity in the praline’s nougat filling compared to the chocolate shell (Figure 1). Over time, these components migrate into and through the shell and finally facilitate the formation of fat bloom on the shell’s surface. This migration and its time scale have been seen as increasing MRI signal intensity in the praline’s shells aging time (Figure 2). This amounted to several weeks and is also known to be a function of chocolate composition, as well as of environmental parameters [19]. In more detail, the preparation and production steps of the chocolates and pralines influence the fat blooming. In Figure 2, this fact is evident on the praline’s bottom: the praline was covered with two chocolate layers providing a migration barrier, which is overcome only at a later time.

Indeed, the fat bloom on the pralines with hazelnut-based fillings shows a significant content of hazelnut oil components, although there was no hazelnut oil contained in the raw chocolate shell. Therefore, migration of unsaturated TAG happened from the filling through the shell to the fat bloom. The identification of the TAG in fat bloom is complemented by the quantification via HPLC–MS/MS. A comprehensive and quantitatively detailed description of chemical composition of fat bloom could finally be achieved for the pralines.

Finally, fat bloom from the commercially available chocolates containing no nuts (yoghurt) show no significantly different composition to cocoa butter. We conclude that the fat bloom is specific to the chocolate/praline composition. The bloom formation itself does not necessarily enrich the small amounts of more unsaturated TAG (such as POO) found in cocoa butter. In chocolates with hazelnut-based fillings, fat bloom is mediated by hazelnut oil, which migrates and contributes to the fat bloom in the form of OOO, LXX, and LXL. The latter are not present in cocoa butter but are in hazelnut oil.

In summary, the NMR spectra of cocoa butter and of fat bloom differ mainly in the concentration of OOO, with a melting temperature below room temperature. OOO is thus expected to be a rather mobile component and is also responsible for the increasing intensity of chocolate shells in the MR images. TAGs containing L instead of O (for example OLO, OLL or LLL) behave similarly to that of OOO.

## 4. Materials and Methods

### 4.1. Samples

Two different types of samples have been investigated:

- First, self-made batches of chocolate pralines have been produced, while varying the manufacturing parameters. The nut-based fillings contained 50% sugar, 39% hazelnuts, 8% cocoa butter and 3% free fat cocoa powder resulting in a total fat content of 42.5%. The varied process parameters (Table 2) during the production of the nut fillings have been hazelnut roasting time (10, 30 and 60 min), grinding duration and finesse of the mixture of hazelnut, sugar, and cocoa powder (finely, medium and coarse ground), as well as the mixing time (10, 30 and 60 min) and mixing temperature of the completed filling, including cocoa butter (40, 50 and 60 °C). For the preparation of the nut-based filling, the hazelnuts were roasted in the oven (Küppersbusch Hausgeräte GmbH, Essen, Germany) at 145 °C convection. The roasted hazelnuts were pre-crushed for 1 min using a Thermomix TM6 (Vorwerk, Wuppertal, Germany) and ground together with sugar and cocoa powder using a three-roller mill (SDY200, Bühler Group, Uzwil, Switzerland) once (for the medium-ground nut filling) or twice (for the finely ground nut fillings). Pre-tempered cocoa butter was added and mixed using a Thermomix with different duration and temperatures. The nougat pastes were filled in light-protected containers at 20 °C. The dark chocolate shells were kindly provided by August Storck. Each praline shell was filled with 5 g nut filling and covered with two layers of 45 °C pre-tempered dark chocolate. The filled chocolates were stored overnight at 4 °C and then at 20 °C for several weeks, and exemplars were taken out from time to time (fresh, from 1 week up to 16 weeks) to be measured by MRI. To measure the composition by NMR spectroscopy and LC–MS, the nougat filling, chocolate shell and the resulting fat bloom were separated from each other after the removal time and analysed individually.
- The second batch of fat bloom was obtained for commercially available chocolates, in particular yoghurt and nougat chocolates of a German producer. These chocolates were stored at 20 °C for over a year until harvesting of the fat bloom. NMR spectroscopy revealed the chemical composition.

For qualitative analysis of TAG, 17 mg each of hazelnut oil, cocoa butter and 34 mg of chocolate coating and nougat were dissolved in 1 mL deuterated chloroform, centrifuged, and filtered for NMR analysis. Then, 100 uL of this solution was diluted with 900 uL isopropanol for the LC–MS analysis.

For quantitative and qualitative analysis of FA and TAG, the fat bloom samples were first measured by NMR after being dissolved in 600 µL of deuterated chloroform, centrifugation, and transfer to 5 mm NMR tubes.

After NMR measurements, the tubes were transferred into 10 mL glass vials. Each NMR tube was washed with 500 µL isopropanol three times. The wash fractions for each tube were collected in the vial with the corresponding sample. Each sample was evaporated to dryness with nitrogen gas and the dry matter was weighted before reconstitution with 5 mL of isopropanol. 1 mL of each sample was transferred to an HPLC vial for chromatography and subsequent MS.

### 4.2. Preparation of Calibration Standards

FA standards are weighed into a 20 mL flask and filled up to the calibration mark with isopropanol. Compounds, masses and concentrations are listed in Table 7.

Calibration standards were prepared from this stock standard in a working concentration range between 20 ng (Myristic acid) and 3770 ng (Margaric acid), injected on the column with an injection volume of 10 µL. Linear regression coefficients for the calibration curves for the target analytes were between 0.999 and 0.989.

TAG standards are weighed into a 20 mL flask and filled up to the calibration mark with isopropanol. Compounds, masses and concentrations are listed in Table 8.

Calibration standards were prepared from this stock standard in a working concentration range between 40 ng (OOO) and 1400 ng (SOS), injected into the column with an injection volume of 1 µL. Regression coefficients for the logarithmic calibration curves for the target analytes were between 0.977 and 0.952.

### 4.3. MRI

^1^H images of the self-made pralines were measured on a super wide bore 200 MHz Avance HD III spectrometer equipped with a micro 2.5 gradient and a MICWB 40, 25 mm birdcage. The maximum gradient amplitude was thus 1.5 T/m. The software ParaVision 6.0.1 was used for image data acquisition and processing. Pralines in plastic wraps were imaged by RARE (Rapid Acquisition with Relaxation Enhancement) with the acquisition parameters in Table 9. Care has been taken that the pralines and their fat bloom were not damaged.

**Table 9:** Acquisition parameters of MRI experiments.

MRI Parameter	
Pulse sequence	RARE
Repetition time [s]	1
Echo time [ms]	2.24
RARE-factor [-]	1
Field of view (*x*, *y*) [mm × mm]	26 × 20
Data matrix size (*x*, *y*)	128 × 128
Voxel size (*x*, *y*) [µm × µm]	203 × 156
Slice thickness (*z*) [mm]	1
Number of averages [-]	128
Measurement time [min]	273

### 4.4. NMR Spectroscopy

NMR experiments were performed on a Bruker Avance Neo Spectrometer at 800 MHz ^1^H frequency, equipped with a cryogenically cooled inverse H/C/N probe (CP TCI). For full characterization we acquired ^1^H 1D, ^13^C-APT (Attached Proton Test experiment, jmod), HSQC, HMBC (Heteronuclear Multiple Bond Correlation), and HSQC-TOCSY spectra with three different mixing times. For comparative analysis, we only acquired ^1^H 1D and ^13^C-APT spectra (Table 10).

^1^H and ^13^C spectroscopy, also in the form of 2D spectroscopy, have been performed on the dissolved fat bloom to reveal the chemical composition in detail.

### 4.5. HPLC–MS

#### 4.5.1. Chemicals

Methanol “super gradient grade” and Isopropanol “hyper grade for LC–MS” were from Merck, Darmstadt, Germany. Water was produced with a Millipore water purification system from Merck, Darmstadt, Germany. Ammonium formate and lithium formate were from Merk, Darmstadt, Germany. TAG standards were from Biomol GmbH, Hamburg, Germany. Commercially available FA standards were used.

#### 4.5.2. HPLC

Chromatography was performed on an Agilent 1260 HPLC system equipped with a quaternary pump, autosampler, column oven and diode array detector (Agilent, Waldbronn, Germany). The separation was carried out on a 150 × 4.6 mm Waters XSelect HSS C18 column with a particle size of 3.5 µm from Waters, Eschborn, Germany. Injection volume was 10 µL for FA analysis and 1 µL for the analysis of TAG. The column temperature was set to 45 °C, and the flow rate was 0.5 mL/min. The mobile phase was A: 25% water in methanol (5 mM ammonium formate) and B: isopropanol. The elution gradient was: 0 min, 80% A; 35 min, 0% A; 40 min, 0% A; 41 min, 80% A; 50 min, 80% A.

#### 4.5.3. Mass Spectrometry

The outlet of the HPLC was connected to an electrospray ion source operated in negative ionization mode for the detection of FA and in positive ionization mode for the detection of TAG. The mass spectrometer was a MicroTOF-QII from Bruker Daltonic, Bremen, Germany. Mass spectra were acquired from 50 to 1000 *m*/*z* for FA and 50 to 1300 *m*/*z* for TAG. Calibration of the spectrometer was carried out after infusion of sodium acetate at the beginning of the chromatography. In MS/MS operation, the instrument parameters were the same as in the full scan MS mode, except collision energy, which was set between 40 eV and 50 eV (depending on *m*/*z*). Nitrogen was used as collision gas.

## 5. Conclusions

Fat bloom on chocolate is a result of fat/oil migration, and the detailed chemical composition depends on the fat composition, which is given in the form of triacylglycerides (TAGs). TAGs are classified into unsaturated and saturated TAGs, with their specific molecular mobility, depending on the fatty acids. The substitution position of a specific fatty acid on the glyceride’s OH is often unknown, which was successfully addressed by combined ^1^H, ^13^C 1D and 2D NMR spectroscopy. The combination of MRI, NMR spectroscopy, and HPLC–MS/MS has allowed determination of the fat bloom composition. MRI revealed the migration of TAG from the filling though the shell to the crystalline fat bloom, which is driven by the concentration gradients and takes place at the time scale of weeks. NMR spectroscopy revealed the chemical composition of fat bloom on self-made pralines and commercially available chocolates, which reflects the chemical composition of the constituents and their migration. Finally, MS allowed quantification of the TAGs found in the fat bloom by referencing to standards. In summary, it was found that unsaturated TAGs of hazelnut oil migrate and form mixed crystals with TAGs from the shell in the fat bloom in a cooperative process.

## Figures and Tables

**Table 1:** Typical crystal form α, β^(^′^,^″^)^, γ and ranges of melting temperature of some of the TAGs contained in chocolate or fillings, together with references.

Compound	Crystal Forms and Melting Ranges	Aggregate State at 20 °C	References
POS	sub α-3 (-), α-2 (18.2 °C), β′-2 (24.5 °C), β′-3 (33 °C), β-3 (37.4–38 °C)	solid	[6,7,8]
SOS	γ (23–23.5 °C), α (29–30 °C), β″ (36–37.5 °C), β′ (41.5 °C), β (43–43.8 °C)	solid	[9,10]
POP	γ (12 °C), α (21.5 °C), β″ (29 °C), β′ (35 °C), β (37.5 °C)	solid	[10]
OOO	γ (−32 °C), α (−12 °C), β (4.9 °C)	liquid	[11,12]
PLP	-	solid	
POL	sub-α-2 (-), α-2 (-), β′-3 (-)	solid	[13]
LOO	α-2 (-), β′-2 (-)	liquid	[13]
POO	α (−4 °C), β (18.2–19 °C)	liquid	[14]
SOO	sub-α-2 (-), α-2 (-), β′-3 (-)	solid	[13]

**Table 2:** Overview of the different preparation parameters of pralines. The pralines (P with a number) differ in their nut filling. During the production of the nut filling, the roasting time, the mixing time, the mixing temperature and the degree of grinding were varied. P6, P8 and P10 are identical to P1 and were therefore not included in the paper.

Sample	Roasting Time (min)	Mixing Time (min)	Mixing Temperature (°C)	Degree of Grinding
P1	30	60	40	fine
P2	30	60	50	fine
P3	30	60	60	fine
P4	30	10	40	fine
P5	30	30	40	fine
P7	10	60	40	fine
P9	30	60	40	fine
P11	60	60	40	medium
P12	30	60	40	coarse

**Table 3:** Compilation of tentatively identified TAGs and the corresponding FA bound to them in starting material of praline production and fat bloom on the samples (RT: retention time, ID: identification, L’: linolenic acid, n.a.: no data available). TAG in bold letters: reference standards available. TAG names do not indicate positional location of FA in the TAG. The table is sorted according to degree of unsaturation, starting with the highest DBE value.

RT (min)	*m*/*z*	ID	Sum Formula TAG	DBE	ID	Sum Formula FA	DBE	ID	Sum Formula FA	DBE	ID	Sum Formula FA	DBE
30.9	894.756	LLL’	C_57_H_96_O_6_	10	L	C_18_H_32_O_2_	3	L	C_18_H_32_O_2_	3	L’	C_18_H_30_O_2_	4
31.5	896.773	LLL	C_57_H_98_O_6_	9	L	C_18_H_32_O_2_	3	L	C_18_H_32_O_2_	3	L	C_18_H_32_O_2_	3
32.7	898.786	LOL	C_57_H_100_O_6_	8	L	C_18_H_32_O_2_	3	L	C_18_H_32_O_2_	3	O	C_18_H_34_O_2_	2
32.7	872.770	LPL	C_55_H_98_O_6_	7	L	C_18_H_32_O_2_	3	L	C_18_H_32_O_2_	3	P	C_16_H_32_O_2_	1
33.3	900.803	OLO	C_57_H_102_O_6_	7	O	C_18_H_34_O_2_	2	O	C_18_H_34_O_2_	2	L	C_18_H_32_O_2_	3
31.0	918.813		C_57_H_104_O_7_	6	O	C_18_O_34_O_2_	2	O	C_18_O_34_O_2_	2	O	C_18_O_34_O_3_	2
33.3	874.787	POL	C_55_H_100_O_6_	6	O	C_18_H_34_O_2_	2	L	C_18_H_32_O_2_	3	P	C_16_H_32_O_2_	1
33.9	902.819	**OOO**	C_57_H_104_O_6_	6	O	C_18_H_34_O_2_	2	O	C_18_H_34_O_2_	2	O	C_18_H_34_O_2_	2
34.2	930.848		C_59_H_108_O_6_	6	O	C_18_H_34_O_2_	2	O	C_18_H_34_O_2_	2	paulinic acid	C_30_H_38_O_2_	2
35.0	932.864		C_59_H_110_O_6_	5	n.a.	n.a.	n.a.	n.a.	n.a.	n.a.	n.a.	n.a.	n.a.
33.1	822.756		C_51_H_96_O_6_	5	n.a.	n.a.	n.a.	n.a.	n.a.	n.a.	n.a.	n.a.	n.a.
33.3	876.8043	POO	C_55_H_102_O_6_	5	O	C_18_H_34_O_2_	2	O	C_18_H_34_O_2_	2	P	C_16_H_32_O_2_	1
33.7	876.8043	PLS	C_55_H_102_O_6_	5	P	C_16_H_32_O_2_	1	L	C_18_H_32_O_2_	3	S	C_18_H_36_O_2_	1
34.3	904.8357	SOO	C_57_H_106_O_6_	5	O	C_18_H_34_O_2_	2	O	C_18_H_34_O_2_	2	S	C_18_H_36_O_2_	1
32.4	894.811		C_55_H_104_O_7_	4	n.a.	n.a.	n.a.	n.a.	n.a.	n.a.	n.a.	n.a.	n.a.
33.3	848.771	PLP	C_53_H_98_O_6_	4	P	C_16_H_32_O_2_	1	P	C_16_H_32_O_2_	1	L	C_18_H_32_O_3_	2
33.9	850.788	**POP**	C_53_H_100_O_6_	4	P	C_16_H_32_O_2_	1	P	C_16_H_32_O_2_	1	O	C_18_H_34_O_2_	2
34.6	878.820	**POS**	C_55_H_104_O_6_	4	P	C_16_H_32_O_2_	1	O	C_18_H_34_O_2_	2	S	C_18_H_36_O_2_	1
35.3	906.849	**SOS**	C_57_H_108_O_6_	4	S	C_18_H_36_O_2_	1	S	C_18_H_36_O_2_	1	O	C_18_H_34_O_2_	2
35.8	934.881	OSA	C_59_H_112_O_6_	4	O	C_18_H_34_O_2_	2	S	C_18_H_36_O_2_	1	arachidic acid	C_20_H_40_O_2_	1

**Table 4:** Distribution of TAGs in the starting materials and praline samples based on the ion count integral normalized to the highest integral of OOO present in nougat. The table is sorted according to degree of unsaturation starting with the highest DBE value.

RT (min)	*m*/*z*	ID	[M+NH_4_]^+^	Hazelnut Oil	Nougat	Chocolate Shell	Cocoa Butter	P1	P2	P3	P4	P5	P7	P9	P11	P12
30.9	894.756		C_57_H_100_NO_6_	2%	0%	0%	0%	0%	0%	0%	0%	0%	0%	0%	0%	0%
31.5	896.773		C_57_H_102_NO_6_	8%	6%	0%	0%	1%	0%	1%	0%	1%	1%	1%	0%	1%
32.7	898.786		C_57_H_104_NO_6_	13%	18%	0%	0%	2%	1%	2%	1%	2%	2%	2%	2%	2%
32.7	872.770		C_55_H_102_NO_6_	4%	4%	0%	0%	0%	0%	0%	0%	0%	1%	1%	0%	1%
33.3	900.803		C_57_H_106_NO_6_	39%	51%	0%	0%	5%	5%	6%	5%	7%	7%	7%	5%	6%
31.0	918.813		C_57_H_108_NO_7_	0%	3%	0%	0%	0%	0%	0%	0%	0%	0%	0%	0%	0%
33.3	874.787		C_55_H_104_NO_6_	9%	12%	1%	1%	2%	2%	2%	1%	2%	2%	2%	2%	2%
33.9	902.819	OOO	C_57_H_108_NO_6_	88%	100%	1%	1%	13%	10%	13%	10%	15%	14%	13%	11%	12%
34.2	930.848		C_59_H_112_NO_6_	1%	1%	0%	0%	0%	0%	0%	0%	0%	0%	0%	0%	0%
33.3	876.804		C_55_H_106_NO_6_	21%	29%	0%	0%	0%	0%	0%	0%	0%	0%	0%	0%	0%
33.7	876.804		C_55_H_106_NO_6_	0%	0%	11%	12%	8%	7%	9%	7%	9%	9%	10%	9%	11%
33.3	848.771		C_53_H_102_NO_6_	0%	3%	4%	5%	2%	2%	2%	2%	2%	3%	3%	3%	4%
35.0	932.864		C_59_H_114_NO_6_	1%	1%	0%	0%	0%	0%	0%	0%	0%	0%	0%	0%	0%
34.3	904.845		C_57_H_110_NO_6_	16%	22%	8%	4%	4%	4%	4%	3%	4%	5%	5%	5%	5%
32.4	894.811		C_55_H_108_NO_7_	0%	0%	1%	1%	1%	1%	1%	1%	1%	1%	1%	1%	1%
33.1	822.756		C_51_H_100_NO_6_	0%	0%	0%	1%	0%	1%	1%	0%	0%	1%	1%	1%	1%
33.9	850.788	POP	C_53_H_104_NO_6_	1%	9%	33%	33%	28%	31%	35%	28%	30%	33%	42%	36%	43%
34.6	878.820	POS	C_55_H_108_NO_6_	2%	33%	57%	56%	51%	51%	59%	46%	53%	57%	65%	58%	66%
35.3	906.849	SOS	C_57_H_112_NO_6_	1%	23%	41%	39%	31%	30%	34%	27%	34%	37%	41%	34%	38%
35.8	934.881		C_59_H_116_NO_6_	0%	12%	0%	11%	8%	7%	9%	7%	9%	9%	10%	9%	11%

**Table 5:** Quantitative determination of FAs in starting material and in fat bloom of the praline samples as amount present in (µg) (n.d.: not detected).

Sample	L	P	O	S	Behenic Acid	Lignoceric Acid	Margaric Acid	Myristic Acid	Sample Weight
P1	1	12	9	4	n.d.	n.d.	n.d.	n.d.	6050
P2	2	9	7	3	n.d.	n.d.	n.d.	n.d.	4637
P3	2	10	9	2	n.d.	n.d.	n.d.	n.d.	6900
P4	1	8	8	1	n.d.	n.d.	n.d.	n.d.	5150
P5	1	13	10	6	n.d.	n.d.	n.d.	n.d.	6237
P7	3	14	13	5	n.d.	n.d.	n.d.	n.d.	5712
P9	3	14	11	6	n.d.	n.d.	n.d.	n.d.	6850
P11	4	17	13	8	n.d.	n.d.	n.d.	n.d.	6237
P12	3	16	12	8	n.d.	n.d.	n.d.	n.d.	7187
hazelnut oil	n.d.	n.d.	1	n.d.	n.d.	n.d.	n.d.	n.d.	1700
cocoa butter	3	10	9	7	n.d.	n.d.	n.d.	n.d.	1700
chocolate shell	2	9	7	6	n.d.	n.d.	n.d.	n.d.	1700
nougat filling	8	13	22	8	n.d.	n.d.	n.d.	n.d.	1700

**Table 6:** Amounts of target TAG relative to sample weight which are present in the fat bloom of praline samples.

Sample	% POP	% OOO	% POS	% SOS	Sum
P1	12%	6%	35%	15%	68%
P2	19%	6%	47%	19%	90%
P3	15%	5%	45%	16%	81%
P4	14%	6%	33%	15%	67%
P5	13%	6%	37%	17%	74%
P7	17%	7%	49%	22%	94%
P9	22%	5%	62%	22%	112%
P11	17%	5%	46%	17%	86%
P12	23%	5%	61%	19%	107%

**Table 7:** Preparation FA stock standard.

Compound	CAS	Sum Formula	Molar Mass (g/mol)	[M−H]^−^	Concentration (mg/mL)
Myristic acid	544-63-8	C_14_H_28_O_2_	228.209	227.202	0.26
P	57-10-3	C_16_H_32_O_2_	256.240	255.233	0.65
Margaric acid	506-12-7	C_17_H_34_O_2_	270.256	269.249	0.75
L	60-33-3	C_18_H_32_O_2_	280.240	279.233	0.54
O	112-80-1	C_18_H_34_O_2_	282.256	281.249	0.64
S	57-11-4	C_18_H_36_O_2_	284.272	283.264	0.32
Behenic acid	112-85-6	C_22_H_44_O_2_	340.334	339.327	0.32
Lignoceric acid	557-59-5	C_24_H_48_O_2_	368.365	367.358	0.28

**Table 8:** Preparation of TAG stock standard.

Compound	CAS	Sum Formula	Molar Mass (g/mol)	[M+NH_4_]^+^	Concentration (mg/mL)
OOO	122-32-7	C_57_H_104_O_6_	885.43	902.82	1.28
POP	2190-25-2	C_53_H_100_O_6_	832.75	850.79	1.39
POS	2190-27-4	C_55_H_104_O_6_	860.78	878.82	1.25
SOS	2846-04-0	C_57_H_108_O_6_	888.81	906.85	1.40

**Table 10:** Acquisition parameters of NMR experiments.

NMR Parameter					
Pulse sequence	zg	jmod	hsqcetgpsp.2	hmbcetgpl3nd	hsqcdiedetgpsisp.2
Repetition time (s)	-	6.4	1.39	1.79	1.89
Acquisition time AQ (s)	4.06	5.4	0.39	0.79	0.39
Carrier offset OnP (^1^H/^13^C) (ppm)	3/-	3/100	3/72	3/94	3/42
Spectral width SW (^1^H/^13^C) (ppm)	20.15/-	-/241	13/128	13/170	13/64
Number of points TD (^1^H/^13^C)	128k/-	-/512k	8k/1k	16k/1k	8k/4k
J coupling nJ_CH (Hz)	-	145	145	8	145
TOCSY-mixing time (ms)	-	-	-	-	30/50/100
Number of scans NS (-)	1	6400	2	2	2
Measurement time (min)	0.1	687	49	67	280

## Data Availability

The data including the corresponding parameter files are available on request to the authors.
